# An analysis of the effectiveness of reflective learning through watching videos recorded with smart glasses—With multiple views (student, patient, and overall) in radiography education

**DOI:** 10.1371/journal.pone.0296417

**Published:** 2024-01-02

**Authors:** Kenzo Muroi, Shinsuke Kyogoku, Yasuaki Sakano, Hajime Sakamoto, Kazuma Nakazeko, Kazuya Koyama, Issei Fukunaga, Kensuke Hori, Kumiko Kotake, Shuko Nojiri, Miwa Sekine, Yuji Nishizaki, Hiroyuki Daida

**Affiliations:** 1 Department of Radiological Technology, Faculty of Health Science, Juntendo University, Bunkyo-ku, Tokyo, Japan; 2 Home Care Nursing, Faculty of Nursing, Nara Medical University, Kashihara, Nara, Japan; 3 Medical Technology Innovation Center, Juntendo University, Bunkyo-ku, Tokyo, Japan; 4 Division of Medical Education, Faculty of Medicine, Juntendo University, Bunkyo-ku, Tokyo, Japan; University of Pisa, ITALY

## Abstract

The Objective Structured Clinical Examination (OSCE) is designed to assess medical students’ skills and attitude competencies before clinical practice. However, no method of reflective learning using video-based content has been used in OSCE education. This study aimed to confirm whether using smart glasses-based educational content is effective for OSCE reflective learning using multiple views (patient, student, and overall). This educational intervention study included a control group exposed to the traditional learning method and an intervention group exposed to a learning method incorporating smart glasses. Participants were 117 (72 in the control group and 45 in the intervention group) third-year radiological technology students scheduled to take the OSCE and 70 (37 in the control group and 33 in the intervention group) who met the eligibility criteria. Mock OSCEs were administered before and after the educational intervention (traditional and smart glasses-based education) to investigate changes in scores. After the educational intervention, a self-reported comprehension survey and a questionnaire were administered on the effectiveness of the video-based content from different views for student reflective learning. Unexpectedly, the OSCE evaluation score after the preliminary investigation significantly increased for the smart glasses control group (0.36±0.1) compared to the intervention group (0.06±0.1) setting up the radiographic conditions (x-ray center and detector center; p = 0.042). The intervention group’s lower score in the mock OSCEs may have been due to the discomfort of wearing the smart glasses to perform the radiography procedure and their unfamiliarity with the smart glasses, which may have affected their concentration. The findings suggest that smart glasses-based education for OSCEs can be improved (e.g., being easy to handle and use and trouble-free).

## Introduction

The main purpose of an Objective Structured Clinical Examination (OSCE) is to assess medical students’ skills and attitudes prior to clinical practice [1–3], and it can also serve as an educational tool for healthcare professionals [4, 5]. In the field of radiological technology, OSCEs have been used for educational purposes in recent years [6–8]. The main clinical work of radiology technologists involves tasks related to radiography, and the skills and attitudes that must be acquired prior to clinical training include the capacity to treat patients and operate equipment and radiographic positioning. Accordingly, to confirm that students preparing for clinical training have acquired a certain level of competency regarding these skills and attitudes, they are assessed through the OSCE.

Before taking the OSCE, students must study the OSCE items. For example, in the field of radiography, students learn how to handle radiography equipment, how to set radiography conditions, and how to provide patient care as radiography skills to study radiography work. Faculty members give these technical instructions to students. Students learn through classroom lectures and practical instruction from the faculty. During the practical skills instruction, students receive a verbal explanation of the content from their supervisors and are supposed to reflect on their learning for improvement based on what they remember.

Smart glasses with a video camera that can record video data have become increasingly popular recently. In fact, researchers have reported on the use of these devices in education in the fields of medicine, nursing, and emergency medical services [9, 10]. The assumption behind their use is that by recording students’ activities during the OSCE, students can then use these recorded data as information for pre-OSCE and reflective learning. Hence, using these devices may enable innovative learning content through videos and engagement in reflective learning using objective information. This is a form of education that was not available until recently. However, to the best of our knowledge, there is currently no study on educational content related to smart glasses use in OSCE radiology education.

This study aimed to evaluate smart glasses-based educational content from three different views in OSCE practical skills content among radiological technology students. We also attempted to examine the educational effects of reflective learning with this new content. Our examination included three views: student, patient, and overall.

## Materials and methods

### Design

The study design was an educational intervention study. It involved two student groups being exposed to either a traditional learning method (control group) or a learning method with additional educational content using smart glasses (intervention group).

This study was conducted after approval by the Juntendo University Faculty of Health Sciences Ethics Committee for Research and Other Ethical Matters (receipt number: 22–017). When conducting the study, the researcher explained the research to the participants orally and in writing and confirmed their consent by having them sign a consent form. After obtaining consent, anonymous responses were collected from all consenting students. There are no conflicts of interest related to this study.

### Participants, eligibility criteria, and settings

The study was conducted among 117 third-year radiological technology students at Juntendo University who were scheduled to take the OSCE. Juntendo University, founded in 1838, is a health general university and graduate school consisting of eight faculties, four graduate schools, and six affiliated hospitals, with the School of Medicine as its core [11, 12]. The radiological technology students who participated in this study belong to the Faculty of Health Sciences.

The students were divided into eight groups (14–15 students per group) based on their student numbers. In each block, four groups were randomly assigned to either the intervention or control group. The randomization process was carried out by a statistician who was not involved in the intervention. Depending on the availability of smart glasses, there were five control groups (72 participants) and three intervention groups (45 participants).

Students received preliminary instruction before the OSCE, and one group was taught weekly for a total instructional period of eight weeks. The study was conducted from May to June 2022, during which the participants were recruited.

Eligibility for participation in the study was defined as individuals who agreed to participate and were able to complete the OSCE. Exclusion criteria were applied to individuals who could not agree to participate or could not complete the OSCE. In total, 70 participants (37 in the control group and 33 in the intervention group) agreed to complete the questionnaire and successfully completed the OSCE.

### Mock OSCE

**Fig 1:**
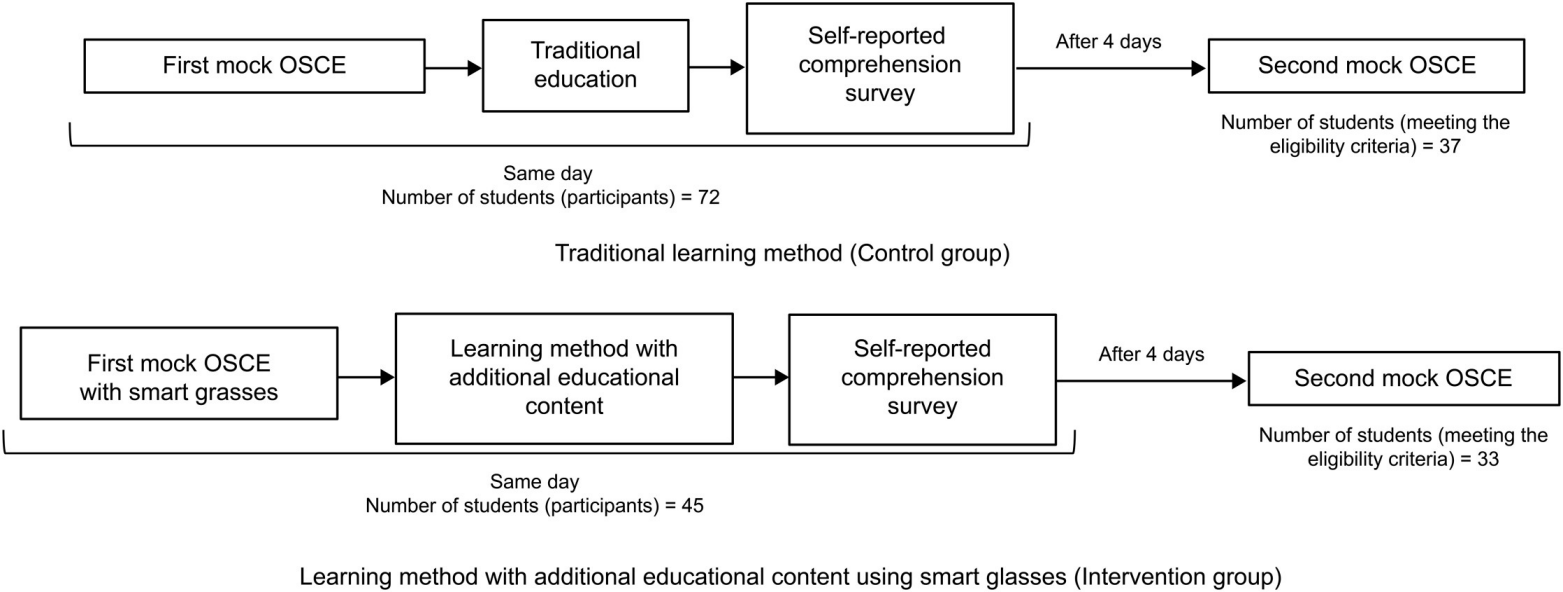
The timing of the mock OSCEs before and after the educational intervention and self-reported comprehension survey.

In the general radiological technology field, radiographic techniques are tested as one of the OSCEs (e.g., chest radiography, body X-ray CT examination, head MRI examination). In this study, we selected chest radiography as the OSCE test item. Fig 1 shows the timing of the administration of the mock OSCEs, educational intervention, and self-reported comprehension survey. The OSCE, educational intervention, and self-report comprehension survey were conducted in the radiography laboratory of the Juntendo University Faculty of Health Sciences, Department of Radiological Technology Practice Building. Students and assessors met during the scheduled OSCE teaching time. Students were briefed on the OSCE by the assessor and then conducted the first mock OSCE. The mock patients were cared for by students who had not previously conducted an OSCE. After the educational intervention (conventional or smart glass education), a self-report comprehension survey was administered. The first OSCE, educational intervention, and comprehension survey were all conducted on the same day. The second mock OSCE was conducted four days after the educational intervention. Students viewed recordings of the first mock OSCE and examined them from three views. The recorded data obtained from filming were stored on a dedicated server and kept in the laboratory. Students viewed the recordings in the laboratory under the supervision of the researcher.

The evaluators were five faculty members who were licensed radiology technologists with at least five years of clinical experience. Each mock OSCE was conducted by two faculty evaluators.

The mock OSCE consisted of 18 evaluation items. These items are listed in Supplement 1. The evaluation was graded on a 4-point scale as follows: *all items were completed* (3 points); *mostly completed* (2 points); *somewhat incomplete* (1 point); and *insufficient* (0 points).

Supplement 1 18 evaluations in the mock OSCE

I. Clean and neat clothing (white coat, pants, shoes, etc.).
II. Hygienic and clean appearance (nails, hair, beard, nose hair, etc.).
III. Setting up the radiographic conditions (1) (radiographic distance).
IV. Setting up the radiographic conditions (2) (X-ray center and detector center).
V. Setting up the radiographic conditions (3) (tube voltage, tube current, and imaging time settings).
VI. Confirmation of patient name and imaging site.
VII. Explanation of changing clothes (obstruction shadow removal, confirming whether the person is wearing any metallic accessory, etc.).
VIII. Positioning (1) (PA chest radiography).
IX. Positioning (2) (remove the shoulder blade from the lung field).
X. Positioning (3) (position of detector and patient).
XI. Positioning (4) (X-ray tube position).
XII. Patient treatment (1) (easy-to-understand voice volume).
XIII. Patient treatment (2) (explanation of examination).
XIV. Patient treatment (3) (breathing cues).
XV. Patient treatment (4) (sincere response).
XVI. Patient treatment (5) (safe and smooth response).
XVII. A word of kindness after the end of the radiographic procedure.
XVIII. Implementation of hand hygiene practices compliant with the WHO guidelines.

### Self-reported comprehension survey

The comprehension survey included nine questionnaire items. These items are listed in S1 Table. The items were rated on a 4-point scale, as follows: *disagree* (4 points), *somewhat disagree* (3 points), *somewhat agree* (2 points), and *agree* (1 point).

Supplement 2 Nine questionnaire at self-reported comprehension survey

I. The preparation (confirmation of radiographic methods) before practical training was sufficient.
II. I was able to understand radiographic positioning through practical training.
III. I was able to master radiographic positioning through practical training.
IV. I was able to understand patient treatment through practical training.
V. I was able to master patient treatment through practical training.
VI. I was able to understand equipment operation through practical training.
VII. I was able to master equipment operation through practical training.
VIII. The radiographic practice was generally satisfactory.
IX. I engaged in practical training with motivation.

### The effectiveness of the video-based content from different views for student reflective learning

An additional self-reported questionnaire was conducted to evaluate the strengths and weaknesses of smart glasses-based education via educational intervention after the second mock OSCE. The video data from the patient, student, and overall views, as well as their combinations, were surveyed. The questionnaire also contained items that asked participants to rate whether the video-based content was effective for specific educational topics, as follows: “the entire radiographic process,” “patient information,” “how you are seen by the patient,” “radiographic positioning,” and “equipment operation.”

### Smart glasses

**Fig 2:**
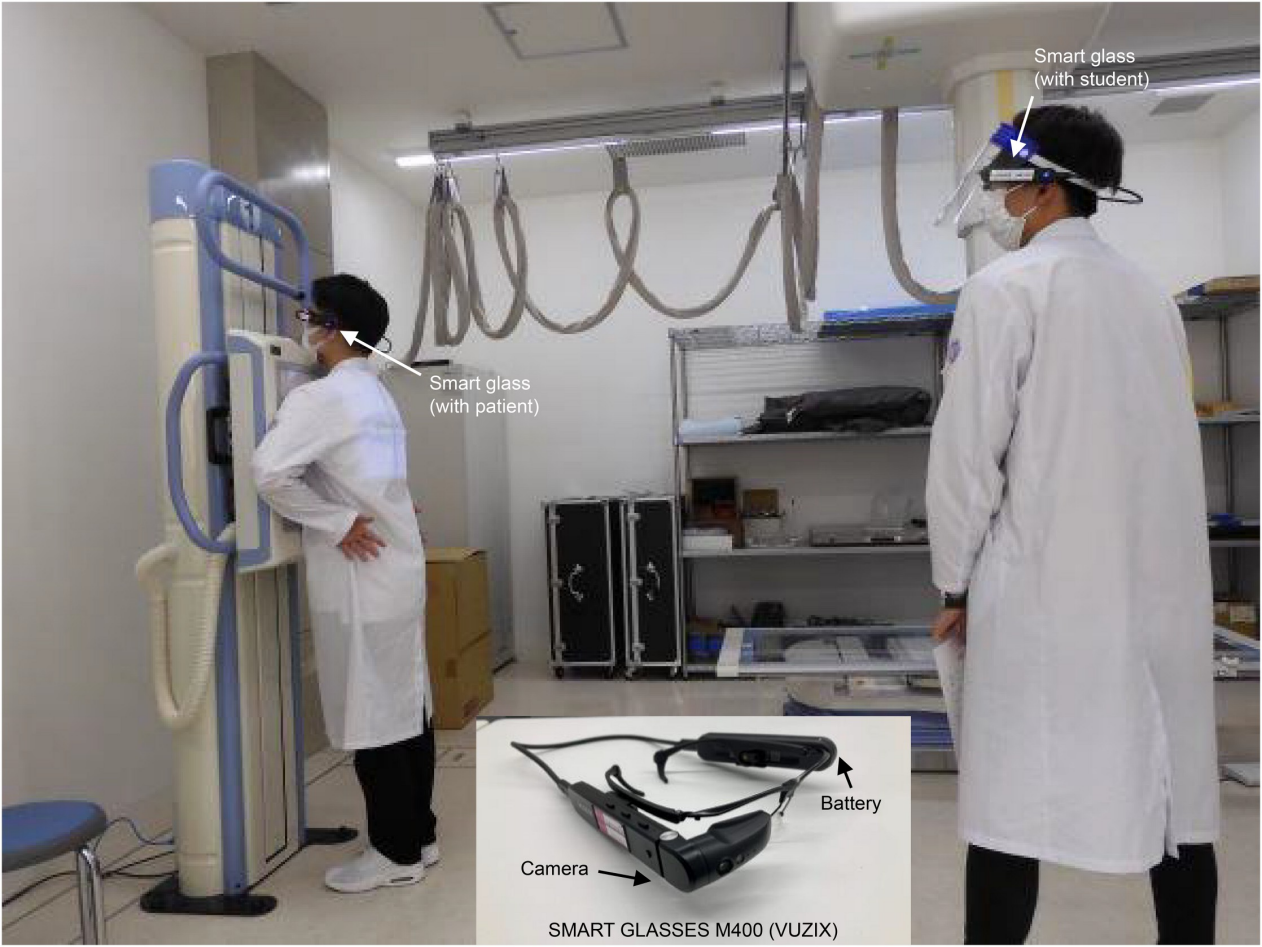
Scene of the mock OSCE with the participants using smart glasses. [Reprinted from Remote NAKAMA under a CC BY license, with permission from AVR Japan Co., Ltd., original copyright 2022].

The smart glasses we used were the SMART GLASSES M400 (VUZIX). They were attached to the head of the mock patient and the student, allowing for capturing images from the view of the front of the head of each person (Fig 2).

**Fig 3:**
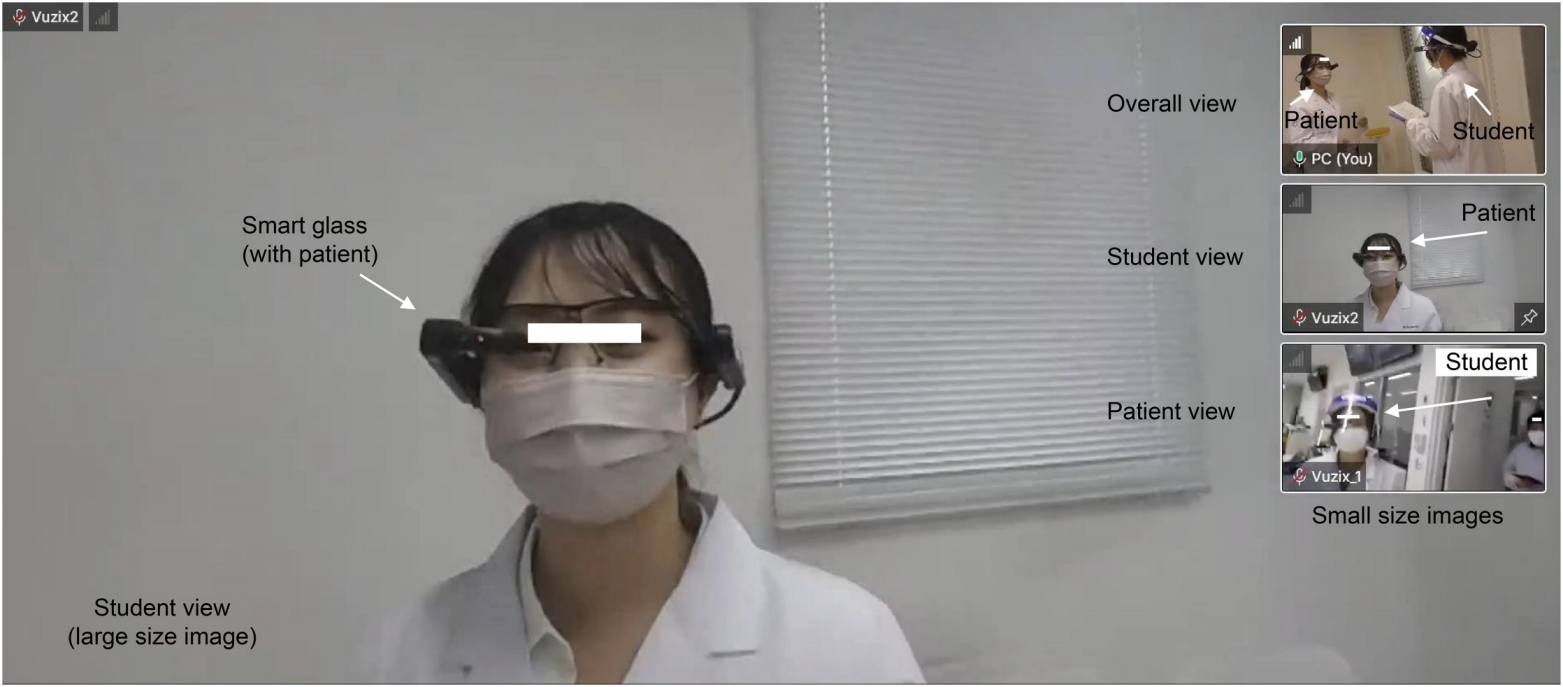
Educational content videos (“overall view,” “student view,” and “patient view”). [Reprinted from Remote NAKAMA under a CC BY license, with permission from AVR Japan Co., Ltd., original copyright 2022].

The compact camera head AG-UCK 20G (Panasonic) and memory card portable recorder AG-UMR 20 (Panasonic) were used as the video camera system for acquiring the video data. By using the online features of the Remote NAKAMA, one can simultaneously observe, on a single screen and in real-time, an image from the mock patient’s view, the student’s view, and the room camera (Fig 3). This screen can be shown to the student group during a mock OSCE. The Remote NAKAMA software requires a general-purpose computer to be run, and we used the notebook PC ASUS GAMING A15 (Model FA506IC). The recording software was OBS Studio version 27.2.4. Remote NAKAMA (AVR Japan Co., Ltd.) was used as the remote working eye system for the wearable device.

### Statistical analysis

We calculated mean scores and standard errors for the first and second mock OSCEs (i.e., before and after preliminary instruction, respectively) to evaluate the changes between the control and intervention groups.

Descriptive analyses were used to examine participants’ basic characteristics. Frequencies and percentages were used for categorical variables, and means and standard error of the mean were used for continuous variables. The Pearson chi-square test was used to determine whether there was a statistically significant difference between the variables. The z-test was used to further investigate the population proportion. The Shapiro–Wilk test and box plots were used to assess the normality of the self-reported comprehension survey. Using the survey results for normality, the Mann–Whitney U test was used to determine the significance of the differences in self-reported comprehension survey scores between the intervention and control groups. Analysis of covariance was conducted to determine the significance of the differences in OSCE scores between the intervention and control groups on score increase, controlling for the OSCE score prior to intervention. Analysis of covariance for the score of OSCE items I and II was not applicable because Levene’s test for equality of variances was violated for these. Subsequently, pairwise comparisons were conducted to compare the intervention and control groups. Statistical significance was set at a p < .050 for each analysis. The p-value threshold for significance was adjusted for multiple comparisons using the Bonferroni correction. Microsoft Excel and SPSS for Windows (IBM) ver.28 were used for data management and data analysis.

## Results

Participants’ basic information is shown in Table 1. Upon comparing the control and intervention groups, we found no significant differences regarding sex, place of origin, school, or grade point average. Table 2 shows the scores for the first and second mock OSCE evaluations. A significant increase can be observed in the scores in the control group (0.36±0.1) compared to those of the intervention group (0.06±0.1) in evaluation item IV: “setting up the radiographic conditions (2) (X-ray center and detector center; p = 0.042).” No significant differences were found in the other items.

**Table 1:** Participants’ characteristics.

	N (%)	Intervention group	Control group	Total	p
**Sex**					
	Male	20 (44.44)	30 (41.67)	50 (42.74)	.768
	Female	25 (55.56)	42 (58.33)	67 (57.26)	
**Hometown**					
	Metropolises^a^	5 (11.11)	6 (8.33)	11 (9.4)	.616
	Other area	40 (88.89)	66 (91.67)	106 (90.6)	
**Graduated from**					
	Public school	27 (60)	47 (65.28)	74 (63.25)	.565
	Private school	18 (40)	25 (34.72)	43 (36.75)	
**Grade point average**		2.73±0.06	2.85±0.05	2.8±0.04	.136

Metropolises: The 23 wards of Tokyo, ordinance-designated city.

**Table 2:** Difference of scores for the mock OSCE evaluations before and after the educational intervention. For assessment item IV: “setting radiographic conditions (2) X-ray center and detector center”, there was a significant increase in scores in the control group (0.36 ± 0.1) compared to the intervention group (0.06 ± 0.1) (p = 0.042). No significant differences were found for the other items.

Analysis of covariance-adjusted comparison	Adjusted				Comparison				Levene’s test for equality of variances
	Intervention group (N = 33)		Control group (N = 37)						
	Mean±SE	95%CI [LL-UL]	Mean±SE	95%CI [LL-UL]	Intervention group—Target group	SE	p	95%CI [LL-UL]	
**I. Clean and neat clothing (white coat, pants, shoes, etc.)**	-	-	-	-	-	-	-	-	.006
**II. Hygienic and clean appearance (nails, hair, beard, nose hair, etc.)**	-	-	-	-	-	-	-	-	.000
**III. Setting up the radiographic conditions (1) (radiographic distance)**	0.28±0.1	[0.08–0.47]	0.5±0.09	[0.31–0.68]	-0.22	0.14	.114	[-0.49–0.05]	.230
IV. Setting up the radiographic conditions (2) (X-ray center and detector center)	0.06±0.1	[-0.15–0.26]	0.36±0.1	[0.16–0.55]	-0.30	0.15	.042	[-0.59–0.01]	.216
V. Setting up the radiographic conditions (3) (tube voltage, tube current, time setting)	0.91±0.13	[0.64–1.18]	0.82±0.13	[0.57–1.07]	0.09	0.19	.644	[-0.28–0.46]	.743
**VI. Confirmation of patient name and imaging site**	0.27±0.07	[0.13–0.41]	0.34±0.07	[0.21–0.47]	-0.07	0.10	.467	[-0.26–0.12]	.807
**VII. Explanation of changing clothes (obstruction shadow removal, confirming whether the person is wearing any metallic accessory, etc.)**	0.49±0.05	[0.38–0.6]	0.39±0.05	[0.29–0.49]	0.10	0.08	.196	[-0.05–0.25]	.335
**VIII. Positioning (1) (PA chest radiography)**	0.32±0.05	[0.22–0.43]	0.36±0.05	[0.26–0.46]	-0.04	0.07	.607	[-0.18–0.11]	.154
IX. Positioning (2) (remove the shoulder blade from the lung field)	0.5±0.09	[0.32–0.69]	0.35±0.09	[0.17–0.52]	0.16	0.13	.225	[-0.1–0.41]	.232
X. Positioning (3) (position of detector and patient)	0.48±0.1	[0.28–0.68]	0.29±0.09	[0.1–0.47]	0.20	0.14	.154	[-0.08–0.47]	.519
XI. Positioning (4) (X-ray tube position)	0.72±0.1	[0.51–0.93]	0.58±0.1	[0.38–0.77]	0.14	0.14	.323	[-0.14–0.43]	.701
**XII. Patient treatment (1) (easy-to-understand voice volume)**	0.63±0.05	[0.53–0.74]	0.68±0.05	[0.58–0.78]	-0.05	0.07	.514	[-0.19–0.1]	.550
XIII. Patient treatment (2) (explanation of examination)	0.42±0.05	[0.32–0.52]	0.44±0.05	[0.34–0.53]	-0.01	0.07	.847	[-0.16–0.13]	.668
XIV. Patient treatment (3) (breathing cues)	0.54±0.08	[0.39–0.69]	0.48±0.07	[0.33–0.62]	0.06	0.11	.559	[-0.15–0.27]	.139
**XV. Patient treatment (4) (sincere response)**	0.49±0.05	[0.4–0.59]	0.44±0.05	[0.35–0.53]	0.05	0.07	.415	[-0.08–0.19]	.255
**XVI. Patient treatment (5) (safe and smooth response)**	0.87±0.07	[0.72–1.01]	0.74±0.07	[0.61–0.88]	0.12	0.11	.251	[-0.09–0.34]	.068
**XVII. A word of kindness after the end of the radiographic procedure**	0.49±0.05	[0.38–0.6]	0.38±0.05	[0.27–0.48]	0.11	0.08	.151	[-0.04–0.26]	.692
**XVIII. Implementation of hand hygiene practices compliant with the WHO guidelines**	1.09±0.09	[0.9–1.27]	0.83±0.09	[0.66–1]	0.25	0.13	.062	[-0.01–0.52]	.523

S1 Table shows the results of the self-reported comprehension survey. No significant differences were found for all items.

S1 Fig showed how students rated their preference for the video-based content from different views for the review learning process. The results show that the combination of the three views had the highest scores, while the student view alone had the lowest scores.

S2 Fig shows how students rated their preference for using smart glasses for specific educational items related to the radiographic process. The results show that “the entire radiographic process” had the highest scores for effectiveness, followed by “patient treatment” and “how you are seen by the patient.”

## Discussion

In this study, we devised new video-based educational content from three views (student, mock patient, and overall views) aimed at effectively promoting reflective learning of OSCE practical skills content among radiological technology students.

Before we conducted this study, we expected that the use of video-based educational content with multiple views would provide objective methods for students to engage in reflective learning after the educational intervention, which would then lead to higher scores on the second mock OSCE and on the comprehension questionnaire. However, some items were rated significantly lower in the intervention group than in the control group. Therefore, the findings contradicted our expectations.

The lower scores for the second mock OSCE in the intervention group may be related to the fact that the students found it uncomfortable to perform the radiographic procedures with the smart glasses on their heads. In addition, students were not accustomed to using the smart glasses, which may have affected their ability to concentrate. Previous studies on the use of ophthalmic wearable devices in medical education have shown their usefulness, such as good image quality and user-friendliness in the field of plastic surgery [13]. In the current study, students in both groups had to operate the X-ray imaging device while managing how they treated mock patients, and the intervention group had to do so while wearing the device. As described above, this may have caused difficulty in movement, which may have, in turn, affected how they treated patients and operated equipment. Meanwhile, the control group may not have faced such hindrances. If this is indeed the case, stakeholders may need to develop a device that does not cause discomfort in students wearing it while performing radiographic procedures.

Regarding the results of the self-reported comprehension survey, the use of the ophthalmic wearable device enabled the students to check the status of the OSCE through video images. However, there were rare occasions when the students were wearing the device during the OSCE but could not access the video because the network was interrupted, and they thus had to deal with the disconnection. The extended time spent resolving these issues may have reduced their focus on the training and understanding of the training content. Moreover, the time that students in the control group spent on the educational intervention time followed the period established by the teachers, whereas the intervention group spent significantly more time on the process because they had to replace the device, check the images from the three cameras, prepare for recording, and troubleshoot any issues. In previous studies regarding the use of ophthalmic wearable devices in medical education, problems such as battery life [14–17], missing video frames [17], and network errors [18] were identified. Addressing these issues may require the devices to be easy to replace and wear.

Regarding examples of the use of wearable technology in medicine, Iqbal et al. [19] discovered that head-mounted displays were utilized in surgery (n = 7), imaging (n = 3), simulation and education (n = 2), and navigation tools (n = 1) [19]. In medical education, a case study demonstrated the use of a visual-based finger-tracking system during transurethral prostatectomy, where surgeons could identify areas of interest that were then live-streamed to trainees and medical students [20]. Another study showed a significant positive correlation between simulator and steering wheel performance in the rehabilitation of spinal cord injury patients. However, the limitations of the head-mounted display resulted in longer stopping times, and some participants experienced simulator sickness during the simulation [21].

Additionally, a report analyzing medical students’ satisfaction and teaching effectiveness found that real-time online clinical skills education using a wearable action camera yielded high levels of educational satisfaction. However, there were some negative comments regarding video quality. Nonetheless, the study indicated that online video education with wearable cameras could be a viable alternative to in-person teaching when classes are unavailable [22]. In previous studies, the instructor wore the wearable device while the learner viewed the video from the instructor’s view. In this study, the educational content could be seen from three views (student, mock patient, and overall), with the learner and mock patient wearing the wearable cameras. This allowed for effective learning through multiple video reflections, which was not possible previously. However, the use of multiple devices also led to complications like device malfunctions, which had been previously noted with wearable devices and did not improve learning satisfaction in some respects. While the use of wearable technology like smart glasses offers the potential for high educational satisfaction, it is essential to address problems encountered by users, such as device handling, stable network environments, and image quality maintenance).

The combination of the three views (mock patient, student, and overall views) showed the highest scores for effectiveness regarding the reflective learning process, while the student perspective alone showed the lowest scores. The OSCE evaluates clinical competence as a healthcare professional in a simulated clinical setting [23, 24]. Therefore, we hypothesized that in a retrospective OSCE to assess students’ clinical competence; it would be effective to visualize a combination of three views: students’ sincere response to a simulated patient (simulated patient perspective), instrument handling (student perspective), and the examination room environment (overall perspective). The student’s perspective would effectively confirm the accuracy of instrument handling in a retrospective study. Wearable cameras have the advantage of recording and visualizing the behavior of the person wearing the camera, as shown in the pilot’s 24-hour usage diary report [25]. We considered the use of wearable cameras to visualize students’ views to be effective for reflective learning in OSCEs. However, the student’s point of view in this study may be captured from a different direction than the student’s actual point of view, depending on the orientation of the head-mounted camera. Therefore, it is not necessarily the same as the student’s view. Therefore, some of the student perspective videos may not be effective for reflective learning.

This study focused on new video-based educational content and examined the learning outcomes from these videos. An essential aspect of this study was the importance of reflective learning from watching videos recorded with smart glasses. Regarding the specific educational items for which the use of the video-based educational content from different views was effective, students deemed them effective for better understanding “the entire radiographic process,” “patient treatment,” and “how you are seen by the patient.” These videos are most effectively obtained through a combination of the “patient perspective,” “student perspective,” and “overall perspective” that students identified as effective videos for reflective learning. From this, it appears that students find videos that allow them to see their movements, the overall flow of the shooting, and how it looks from the patient’s point of view to be effective for reflective learning.

The video information provided in this content allows students to observe their behavior objectively during the OSCE, which was previously impossible. It is also possible to obtain information students cite as effective teaching material for looking back and learning from the OSCE. Based on the above, we considered that reflective learning with this content could provide effective information in OSCE education.

However, the scores for some of the evaluation items and comprehension questionnaire items did not improve because of some specific problems. Specifically, as aforementioned, it is somewhat complicated to handle the ophthalmic wearable device used in this study because of its multifunctional nature; the device is rather large and may be uncomfortable to wear for some students. Moreover, the process of taking out the device from one student and the other wearing it is not smooth.

Regarding study limitations, one is the difference between the evaluators from one mock OSCE evaluation to another. One of several problems in this study was the lack of human resources. The mock OSCEs were evaluated by two faculty members. The mock OSCE was conducted once a week over eight weeks. This required the cooperation of a total of 16 faculty members. Although it would be optimal for the same faculty member to evaluate all mock OSCEs, it was difficult for the same two faculty members to evaluate all eight weeks because they also had other academic duties. Next, it is desirable to standardize the evaluation criteria for mock OSCEs with more detailed content. Currently, the evaluation criteria are generalized within the university but may differ from those in other schools. Therefore, generalization of results may not be guaranteed. In addition, since the OSCE survey covered 117 students and the number of test sites and evaluators was limited, it was not possible to conduct the survey on the same day for all eligible students. This may have affected the evaluator’s instructions. For the same reason, OSCE instruction could not be conducted on the same day and was spread over eight weeks; as with the OSCE evaluation, it would be desirable to use a more detailed and uniform instructional methodology to ensure uniformity. A limitation of the study design was the difficulty in designing the study as a cluster randomized controlled study.

Finally, in the study, the intervention group should have received training on how to handle smart glasses. The reason for not providing training sessions was time constraints, as there was a need to conduct an OSCE for a total of 117 participants (45 in the intervention group and 72 in the control group) within a limited period.

## Conclusions

In the mock OSCE evaluation after the preliminary instruction, unexpectedly, there was a significant increase in the number of points in the control group compared with the intervention group in the setting up of the radiographic conditions regarding the x-ray center and detector center. The intervention group’s lower score on the second simulated OSCE may have been due to the students’ discomfort with wearing the smart glasses to perform the radiography procedure and their lack of familiarity with the smart glasses, which may have affected their ability to concentrate. It was suggested that these problems should be solved by making the device easy to handle and use (e.g., no network interruption).

## Supporting information

S1 Table Results of the self-reported comprehension survey. (DOCX)

S1 Fig Results for the preference of the smart glasses with different views for self-directed student learning according to students. (TIF)

S2 Fig Results for the preference of the smart glasses with different views that were most effective in understanding the radiographic process according to students. (TIF)
