# Supplementary material for: An analysis of the effectiveness of reflective learning through watching videos recorded with smart glasses—With multiple views (student, patient, and overall) in radiography education
Source: PLoS One. 2024 Jan 2;19(1):e0296417. doi: 10.1371/journal.pone.0296417 (PMC10760730; doi:10.1371/journal.pone.0296417)
Supplement: S1 Table — (DOCX) [file pone.0296417.s001.docx]

**Supplementary Table S1. Results of the self-reported comprehension survey.**

|  | **Intervention group (N=33)** | | **Control group (N=37)** | |
| --- | --- | --- | --- | --- |
|  | **mean** | **S.D.** | **mean** | **S.D.** |
| **I The preparation (confirmation of radiographic methods) before practical training was sufficient.** | 2.61 | 0.80 | 2.48 | 0.80 |
| **II I was able to understand [radiographic positioning] through practical training.** | 3.53 | 0.56 | 3.75 | 0.48 |
| **Ⅲ I was able to learn [radiographic positioning] through practical training.** | 3.25 | 0.69 | 3.46 | 0.54 |
| **Ⅳ I was able to understand [patient treatment] through practical training.** | 3.56 | 0.50 | 3.71 | 0.50 |
| **Ⅴ I was able to learn [patient treatment] through practical training.** | 3.11 | 0.62 | 3.44 | 0.57 |
| **Ⅵ I was able to understand [equipment operation] through practical training.** | 3.47 | 0.56 | 3.73 | 0.45 |
| **Ⅶ I was able to acquire [equipment operation] through practical training.** | 3.22 | 0.59 | 3.42 | 0.61 |
| **VIII The radiographic practice was generally satisfactory.** | 3.36 | 0.68 | 3.60 | 0.63 |
| **Ⅸ I engaged in practical training with motivation.** | 3.69 | 0.47 | 3.79 | 0.50 |

**S.D. (standard deviation)**
