# Supplementary material for: An analysis of the effectiveness of reflective learning through watching videos recorded with smart glasses—With multiple views (student, patient, and overall) in radiography education
Source: PLoS One. 2024 Jan 2;19(1):e0296417. doi: 10.1371/journal.pone.0296417 (PMC10760730; doi:10.1371/journal.pone.0296417)
Supplement: Attachment — Submitted filename: Response to reviewer.docx [file pone.0296417.s005.docx]

**Response to Reviewer’s Comments**

Reviewers' Comments to Author:

Reviewer #2:

- This paper focuses on creating content that allows learning from videos captured using smart glasses, featuring footage of students, patients, and overall activities. This study focuses new video-based educational content. The study discusses the learning outcomes derived from these videos. The essential aspect highlighted is the importance of self-learning through watching videos recorded with smart glasses. However, the title, which currently emphasizes the utility of smart glasses, might not clearly convey whether the learning involves wearing smart glasses or simply watching the recorded videos. Therefore, it would be beneficial to emphasize this aspect in both the title and the content.

**Reply:**

Thank you for your comments. As pointed out by you, this study focuses on new video-based educational content and discusses the learning outcomes from these videos. The essential aspect highlighted is the importance of reflective learning through watching videos recorded with smart glasses, which has been analyzed in the study. Therefore, the title has been changed to “An analysis of the effectiveness of reflective learning through watching videos recorded with smart glasses—with multiple views (student, patient, and overall) in radiography education” (please see page 1, lines 2–4).

Additionally, for the purpose of emphasizing that an essential aspect of this study is the importance of reflective learning by watching videos recorded with smart glasses, the following sentences have been added to the discussion section: “This study focused on new video-based educational content and examined the learning outcomes from these videos. An essential aspect of this study was the importance of reflective learning from watching videos recorded with smart glasses.” (please see page 19, lines 336–338).

Finally, we have added “reflective learning” in the introduction section (please see page 4, lines 81) and discussion section (please see page 20, line 350) to emphasize this concept.
